# Using machine learning to identify parenting features prospectively related to callous-unemotional traits from infancy to early adolescence

**DOI:** 10.1017/S0033291726103213

**Published:** 2026-02-18

**Authors:** Y. Paz, S.C. Vogel, P.K. Goh, E.R. Perkins, A. Broussard, N. Huth, A.J. Rosellini, R. Mills-Koonce, M.T. Willoughby, N.J. Wagner, R. Waller

**Affiliations:** 1 University of Pennsylvania; 2 University of Massachusetts Boston; 3 University of Hawai’i at Mānoa; 4 Boston University; 5 University of North Carolina; 6 Research Triangle Institute (RTI International)

**Keywords:** callous-unemotional traits, conduct problems, machine learning, parenting, treatment

## Abstract

**Background:**

Parenting is related to the development of callous-unemotional (CU) traits (i.e. low empathy and restricted guilt), making it an important target of interventions for childhood conduct problems (CPs). However, the relative importance of different parenting features in relation to the development of CU traits remains unclear. This study used machine learning to examine multiple parenting features assessed across infancy and early childhood as predictors of CU traits and CPs in early adolescence.

**Methods:**

Data were from the Family Life Project (*N* = 1,292; 49% female, 41% Black, and 28% below the poverty line). Seventy-four parenting predictors were assessed at eight time points between children aged 6–90 months using parent-reported questionnaires and observer ratings of videotaped interactions and home visits. CU traits and CPs were assessed via parent-reported questionnaires in preadolescence (12–14 years).

**Results:**

Parenting features explained 8.2% of CU traits variability in preadolescence, with top predictors including early sensitive parenting and later behavior management and scaffolding practices. Prediction of CPs was weaker, with parenting explaining 4.5% of the variability.

**Conclusions:**

Results highlight that disruption in close and sensitive early parent–child relationships is relevant to the development of CU traits. Results from the prediction of CPs indicate a more heterogeneous etiology. Findings support targeting parental sensitivity and behavior management within preventative interventions for CU traits and CPs.

## Introduction

Conduct problems (CPs) (e.g. aggression and rule-breaking) are common behavioral concerns in childhood (Merikangas, Nakamura, & Kessler, 2022; Polanczyk et al., 2015). CPs incur significant social and economic costs (Goulter et al., 2024; Rivenbark et al., 2018), increasing lifetime risk for psychiatric conditions, including antisocial personality, major depressive, and substance use disorders (Bevilacqua, Hale, Barker, & Viner, 2018; Kimonis, Paul, & Frick, 2010). Callous-unemotional (CU) traits (i.e. low empathy and lack of guilt) predict greater risk for severe CPs, as well as higher risk for adult psychopathy, violence, and antisocial personality disorder, even accounting for CPs severity (Frick, Ray, Thornton, & Kahn, 2014; Hawes et al., 2017; McMahon, Witkiewitz, & Kotler, 2010; Neo & Kimonis, 2021; Waller et al., 2020). Thus, a better understanding of risk factors for CU traits can inform more effective prevention and treatment efforts to reduce the burden of CPs.

Some early findings on heritability, parenting, and CU traits (Viding, Blair, Moffitt, & Plomin, 2005) suggested that parenting was a less prominent factor in etiological pathways to CPs for children with high CU traits. These findings were sometimes taken to suggest that gold-standard treatments for CPs, which invariably target parenting, are less effective for children with CU traits (Hawes & Dadds, 2005; Oxford, Cavell, & Hughes, 2003; Wootton, Prick, Shelton, & Silverthorn, 1997). Indeed, childhood CU traits are hypothesized to arise from an inherited interpersonal style that renders children less sensitive to socialization cues (Blair, 2017; Viding & McCrory, 2019; Waller & Wagner, 2019). In support of these assertions, meta-analytic work demonstrated that children with CU traits start and end treatments with greater CPs severity than children without CU traits (Perlstein, Fair, Hong, & Waller, 2023).

At the same time, treatments with a parenting component predicted a modest but significant reduction in CU traits (Perlstein et al., 2023). Furthermore, a large literature from the last two decades has unequivocally demonstrated the importance of parenting in risk pathways to CU traits across childhood (for reviews, see Chau, Eapen, Hawkins, & Kohlhoff, 2023; Hawes, Price, & Dadds, 2014; Hyde & Dotterer, 2022; Waller, Gardner, & Hyde, 2013). Moreover, both twin and adoption studies show that parenting practices are modestly associated with CU traits (Hyde et al., 2016; Perlstein et al., 2022; Tomlinson et al., 2022; Waller, Hyde, Klump, & Burt, 2018), alongside evidence for genetic risk (Pezzoli et al., 2024). That is, parenting likely represents a true environmental risk factor for CU traits, even accounting for heritable factors (Perlstein & Waller, 2022).

In general, studies of CU traits have focused on both “negative” (e.g. harsh, coercive) and “positive” (e.g. warm, nurturing, and involved) parenting. Harsh or coercive parenting increases risk for CU traits by desensitizing children to negative environmental cues, exacerbating a fearless or uncaring interpersonal style (Waller & Wagner, 2019). Consistent with the larger CPs literature, harsh parenting also models aggression and coercion as methods of managing conflict (Gershoff, 2002; Waller et al., 2018). At the same time, parenting that is low on warmth or nurturance can stymie children’s experience of positive social interactions, exacerbating their lower social affiliation (Feldman, 2012; Spinrad & Gal, 2018). Likewise, low parental warmth means that children have fewer opportunities to model behavior that is empathic or cooperative, increasing risk for CU traits (Bedford et al., 2017; Facci et al., 2024; Waller & Wagner, 2019). However, questions remain about which aspects of parenting assessed across infancy and early childhood are most relevant to CU traits. Addressing this question can identify the most effective parenting targets to reduce CU traits across developmental stages.

Several limitations of the current literature make these questions challenging to answer. First, prior efforts emphasized broad dimensions of positive or negative parenting in relation to CU traits (Waller et al., 2013). Rarely have studies simultaneously assessed multiple, precise aspects of parenting (i.e. sensitivity and behavior management) and evaluated their relative importance in predicting CU traits, including when positive and negative parenting dimensions are included in the same statistical model (e.g. Waller et al., 2012). Second, prior studies often leverage cross-sectional assessments or short follow-ups, typically within one developmental period (Chau et al., 2023; Hawes et al., 2014; Hyde & Dotterer, 2022; Waller et al., 2013). Thus, we lack knowledge about the relative importance of parenting practices during infancy and toddlerhood, before CU traits can be reliably assessed. Third, prior studies have used both parent reports and observational ratings of parenting behavior, which each bring different strengths and limitations (e.g. shared method variance, report bias, time-consuming, and costly). However, we lack knowledge about which measures, assessed by which method, are most predictive of later CU traits, which could inform future observational and treatment research.

Accordingly, we aimed to identify which reports and observed measures of parenting assessed at numerous times across infancy and childhood were most predictive of CU traits in early adolescence, when CPs can become more persistent, portending risk for lifespan antisocial behavior (Fonagy, 2021; Hinshaw, Lahey, & Hart, 1993). Answering this question required a different approach than prior research, which has relied on regression to isolate unique parenting effects on CU traits. Such methods limit model complexity in terms of the number of predictors that can be simultaneously evaluated (Dwyer, Falkai, & Koutsouleris, 2024), especially for correlated predictors (i.e. measures of parenting). Such methods also fail to account for interactions and nonlinear associations between predictors and outcomes. Finally, even advanced methods, such as network analyses or structural equation modeling, risk overfitting the data and offer limited replicability (Epskamp & Fried, 2018; Whelan & Garavan, 2014). To address these issues, data-driven machine learning (ML) approaches appropriate for prediction analyses are needed, including ML algorithms that build nonparametric decision trees. Within decision trees, the deciding parameter at each split depends on parameters used in preceding splits, thus allowing for the modeling of contingencies between predictors (i.e. interactions) and nonlinear relationships (Breiman, Friedman, Olshen, & Stone, 2017; Venkatasubramaniam et al., 2017).

Thus, in the current study, we adopted an ML approach that enabled a data-driven exploratory test of many correlated parenting variables and identified which features predicted CU traits in preadolescence. We analyzed data from a large sample with numerous repeated parenting assessments across ages 6 months to 7 years. We also tested models with CPs as an outcome to evaluate the specificity of different parenting dimensions in relation to CU traits. Given the breadth of measures, we interpreted findings through established parenting constructs, applying this classification *post hoc* to facilitate interpretation: emotional sensitivity (i.e. warmth, attunement, and responsivity; Macdonald, 1992; Shin, Park, Ryu, & Seomun, 2008), scaffolding (i.e. supporting acquiring new skills; Mermelshtine, 2017), behavior management (i.e. control and response to negative behavior; (Leijten, Melendez-Torres, & Gardner, 2022; Patterson & Fisher, 2002), and parental mastery (i.e. belief in self-efficacy; De Montigny & Lacharité, 2005). We also interpreted findings using the positive versus negative parenting distinction commonly represented in the broader literature and previously applied in the current sample (Barnett et al., 2008; Vernon-Feagans et al., 2013). Prior studies evidence the distinctiveness of these different parenting constructs, both in terms of convergent validity and child outcomes (Grusec & Davidov, 2010). Further, prior studies suggest differential prediction of CU traits versus CPs by different parenting factors (Tomlinson et al., 2022; Waller et al., 2018), highlighting the potential for this research to inform personalized interventions and treatment.

To achieve our study aims, we used an ML random forest algorithm that averaged many trees (Breiman et al., 2017) to assess the importance of different parenting features based on their centrality in predicting CU traits and CPs. While ML analyses are inherently exploratory, we hypothesized that parental sensitivity would emerge as a predictor of CU traits (Bedford et al., 2017; Facci et al., 2024; Goulter, McMahon, Pasalich, & Dodge, 2020), reflecting the centrality of early warmth to the development of early empathy and affiliation (Waller & Wagner, 2019). As children’s behavioral control and self-regulation normatively increase across early childhood, we hypothesized that behavioral management would become particularly predictive of CP (Smith et al., 2014; Speyer, Hang, Hall, & Murray, 2022). Despite limited research, we hypothesized that the fearlessness associated with CU traits might interfere with parents’ sense of efficacy and mastery. In contrast, we did not expect cognitive scaffolding behaviors to rank among the top predictors of either outcome.

## Methods

### Participants

Data were from the Family Life Project, a large longitudinal study (*N* = 1,292, 49% females, 43% Black) administered in rural counties of North Carolina and central Pennsylvania, originally recruited between 2003 and 2004. There were eight assessments between infancy and preadolescence (6, 15, 24, 36, 48, 58, 90, and 154 months; more details in Vernon-Feagans et al., 2013; Willoughby et al., 2013). Parents reported child age, sex, and race, and income-to-needs ratio (Burchinal et al., 2018). Here, we computed an average income-to-needs ratio across the first seven assessments. Recruitment efforts focused on low-income families (28% fell below the poverty line, average income-to-need ratio ≤ 1). We focused on children with available data from the final time point (preadolescence, aged 12–14 years; range, *N* = 861–864). An additional 69 children were excluded because they were missing data for >25% of the parenting predictors described below (cf., Cohen et al., 2020; 2023). The final sample was *N* = 792 for CU traits analyses and *N* = 795 for CP analyses (Supplementary Figure S1). Children excluded from analyses did not differ from the original sample based on race, sex, income-to-needs ratio, CU traits, or CPs (Supplementary Table S1).

### Procedure

Home visits were conducted over two sessions when the children were aged 6, 24, and 36 months and one session at 15, 48, 58, and 154 months. During visits, primary caregivers (the majority of whom were biological mothers) (Willoughby et al., 2013) completed questionnaires, and study staff implemented semi-structured behavioral tasks, which were videotaped for later behavioral coding (Willoughby et al., 2013). After each visit, study staff rated an independent observational measure of caregiver behavior (Berry et al., 2016). All study protocols were approved by the institutional review boards of the University of North Carolina at Chapel Hill, and consent was obtained from all study participants.

### Measures

**CU traits.** We assessed CU traits using parent reports on the 24-item Inventory of Callous-Unemotional Traits (ICU) (Frick, 2004), with items rated on a 4-point scale (0 = *not at all true* to 3 = *definitely true*). Consistent with recommendations (Ray & Frick, 2018), we used a summed score of items (*α* = .87). Early CU traits were measured at 48 months using six items from the Antisocial Process Screening Device items (Dadds, Frost, Fraser, & Hawes, 2005; Wagner et al., 2015). We derived residualized CU traits scores at age 12 years that adjusted for CU traits at 48 months (*r =* .30, *p <* .001).

**CPs**. We assessed CPs using the disruptive behavior disorder symptoms from the Diagnostic Interview Schedule for Children-IV (Shaffer et al., 2000). Parents indicated whether their child had engaged in disruptive behavior, including oppositional defiant and conduct disorder symptoms. We computed a dimensional CPs score as the count of lifetime symptoms (*α* = .87) to be comparable with the ICU, which does not specify a time period (Frick, 2004).

**Parenting predictors**. We assessed parenting behaviors, beliefs, and opinions using report and observational measures collected during seven home visits conducted between ages 6 and 90 months, resulting in 76 parenting features (see Supplementary Table S2 for details and Supplementary Table S3 and Supplementary Figure S2 for correlations). We summarize each measure below (see Supplemental Material for more details).

***Behavioral coding*:** For six of the seven home visits, parents interacted with their child for 10 min (free play, ages 6, 15, and 36 months; semi-structured tasks, such as puzzles or blocks, at 24, 58, and 90 months). All interactions were videotaped for later observational ratings of parenting behavior (i.e. sensitivity, detachment, and cognitive stimulation) using established rating systems that Cox and Crnic (2002) derived from the National Institute of Child Health and Human Development (1999) scheme, which has been extensively described and validated (e.g. Garrett-Peters et al., 2008; Gueron-Sela et al., 2018; Hibel, Granger, Blair, & Cox, 2011; Mills-Koonce et al., 2016; Sulik et al., 2015). Two independent raters rated 30% of the caregiver–child interactions; the cross-rater intraclass correlations (ICCs) for all subscales were > .70 at each time point (ICC, range *=* .71–.90; Supplementary Table S2). Overall, 46 of the 76 parenting predictors (60.5%) were coded from interactions.

***Research assistant ratings***: A trained research assistant completed the Home Observation Measure of the Environment scale (HOME), a widely used and validated observational tool, to evaluate caregiving behaviors during the visit (0 = *no*, 1 = *yes*) (Caldwell & Bradley, 1984). The infancy version was used at ages 6, 15, 24, and 36 months; the early childhood version was used at 58 months; and the first-grade version was used at 90 months (DeJoseph et al., 2021; Hibel et al., 2011; Mills-Koonce, Towe-Goodman, Swingler, & Willoughby, 2022). Given our aims, we focused on subscales evaluating parenting behaviors (e.g. responsivity and stimulation), but not the household environment (e.g. presence of learning materials). Thirteen predictors (17.1%) were from the HOME.

***Parent-reported questionnaires***. At each visit, parents reported on beliefs, opinions, and parenting behaviors using several questionnaires: Parents’ Beliefs about Feelings (Dunsmore & Karn, 2001; 24 months), Parenting Scale (Arnold, O’Leary, Wolff, & Acker, 1993; 48 and 58 months), Parental Beliefs Survey (Luster, Rhoades, & Haas, 1989; 58 months), Coping with Children’s Negative Emotions Scale (Fabes, 1990; 58 months), and Parent Attribution Test (Bugental, 1987; 58 months). Reliabilities are reported in Supplementary Table S2. Two scales with inadequate internal consistency were removed from analyses (Supplementary Material). We included validated subscales from each questionnaire, with 17 predictors (22.4%) from parent report.

***Additional categorizations*:** Out of the 76 predictors, 42 (55.3%) were assessed at ages 0–3 years and 34 (44.7%) at ages 4–7 years. Theoretically, 46 (60.5%) predictors were classified as positive and 30 (39.5%) as negative parenting. Finally, when examining parenting domains, 50 (65.8%) were classified as emotional sensitivity, 13 (17.1%) as behavioral management, 8 (10.5%) as scaffolding, and 5 (6.8%) as parental mastery.

### Analytic strategy

We treated missing data with multiple imputation (Enders, 2022; Jadhav, Pramod, & Ramanathan, 2019) using the missForest algorithm (Stekhoven & Bühlmann, 2012), as random forest algorithms cannot accumulate missing data (Breiman, 2001), and imputations are recommended to preserve sample size (Azur, Stuart, Frangakis, & Leaf, 2011; van Buuren, 2018). Next, we randomly split the sample into training (75% cases) and testing (25% cases) samples. We used the “createDataPartition” function for a balanced split based on the outcome variable (CU traits or CPs). For factor variables, infrequent responses were recoded to the closest level to ensure adequate representation and reduce model instability (Kuhn & Johnson, 2013). We created training and testing samples separately for CU traits and CPs, then generated separate models to predict outcomes. All analyses were conducted in R using the “caret” package (Kuhn, 2008). First, we used recursive feature elimination (RFE) with 74 predictor variables to determine features that predicted outcomes. RFE builds a model using all features, then calculates feature importance, rank-orders features, and then removes features with the least importance based on model performance metrics (i.e. root mean squared error). Once the optimal number of predictors was identified, we created random forest regression models to predict continuous outcomes. We used the training sample to develop and tune models, utilizing the mtry hyperparameter (i.e. the number of random predictors considered at each split within trees), with a five-fold cross-validation repeated five times to enhance robustness. We tested several options for tuning the mtry values (e.g. 5, half of the predictors, and *n*-1 predictors). All other hyperparameters were set to the default criteria (e.g. 500 trees per forest). Model performance was evaluated by *R* ^2^ and ranged from 0 to 1, with higher values indicating better performance. Next, we used *t*-tests to compare the best model after RFE with the best model using the full 74 predictors (chosen after tuning the hyperparameters for the full model). Second, using the best post-RFE model, we calculated variable importance for each predictor in the training sample (i.e. how much prediction accuracy decreases when a predictor’s values are randomly shuffled; Ishwaran, 2007). We generated a variable importance list with visualizations created using ggplot2 (Wickham, 2016). Directionality was determined by computing zero-order correlations between predictors and outcomes. Finally, we applied the algorithm to the testing sample to obtain model accuracy metrics. We ensured independence of the training and testing sets by randomly assigning each participant to one of the sets, selecting all features based on the training set, and using unsupervised imputation methods that do not incorporate label information (see Supplementary Table S5).

## Results

### Predicting CU traits from parenting features

**Figure 1.**
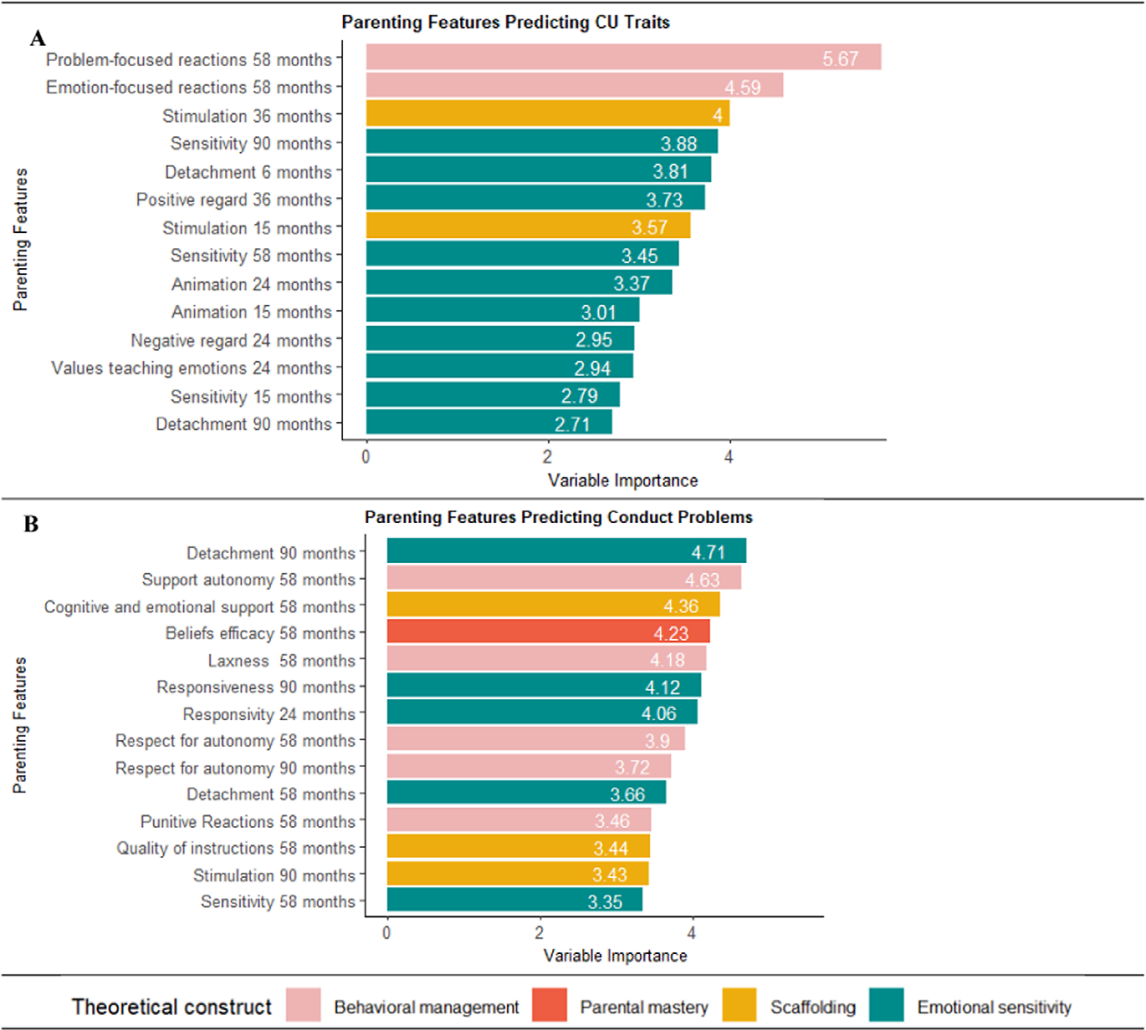
Parenting predictors of CU traits and CPs categorized by different parenting constructs. *Note*: (a) CU traits: See Supplementary Table S5 for the full list of predictors. The top 14 predictors were: (1) Less problem-focused reaction to child negative affect (58 months); (2) Less emotion-focused reaction to child negative affect (58 months); (3) Low cognitive stimulation (35 months); (4) Low sensitivity during interactions (90 months); (5) Detachment during interactions (6 months); (6) Low positive regard toward the child in interaction (35 months); (7) Low cognitive stimulation (15 months); (8) Low sensitivity during interactions (58 months); (9) Less animated behavior (24 months); (10) Less animated behavior (15 months); (11) More negative regard toward the child (24 months); (12) Parents not valuing teaching about emotions (24 months); (13) Low sensitivity during interactions (15 months); and (14) Detachment during interactions (90 months). (b) CPs: See Supplementary Table S6 for the full list of predictors. The top 14 predictors were:(1) Detachment during interactions (90 months); (2) More support for child autonomy (58 months); (3) Low support of cognitive and emotional development (58 months); (4) Parents beliefs that they are unable to influence child development (58 months); (5) More parental laxness (58 months); (6) Low responsiveness (90 months); (7) Responsivity (24 months); (8) and (9) Respect for child autonomy (58 and 90 months); (10) Detachment during interactions (58 months); (11) Punitive reaction to child negative affect (58 months); (12) Quality of instruction (58 months); (13) Less cognitive stimulation (90 months); (14) Low sensitivity during interactions (58 months).

In the training sample, the best model had 71 predictors (mtry = 5), which explained 6.7% of variability in CU traits (standard deviation [SD] = 3.8%). The RFE model did not differ from the full model with 74 predictors (*R* ^2^: *t*(24) = .41, *p* = .68; root mean square error [RMSE]: *t*(24) = −.12, *p* = .90) (Supplementary Table S4). In the testing sample, the model predicted 8.2% of variability in CU traits. For interpretability, we focus on the top 20% (*n* = 14) of predictors (Figure 1a; see Supplementary Table S5 for the full variable importance list).

**Figure 2.**
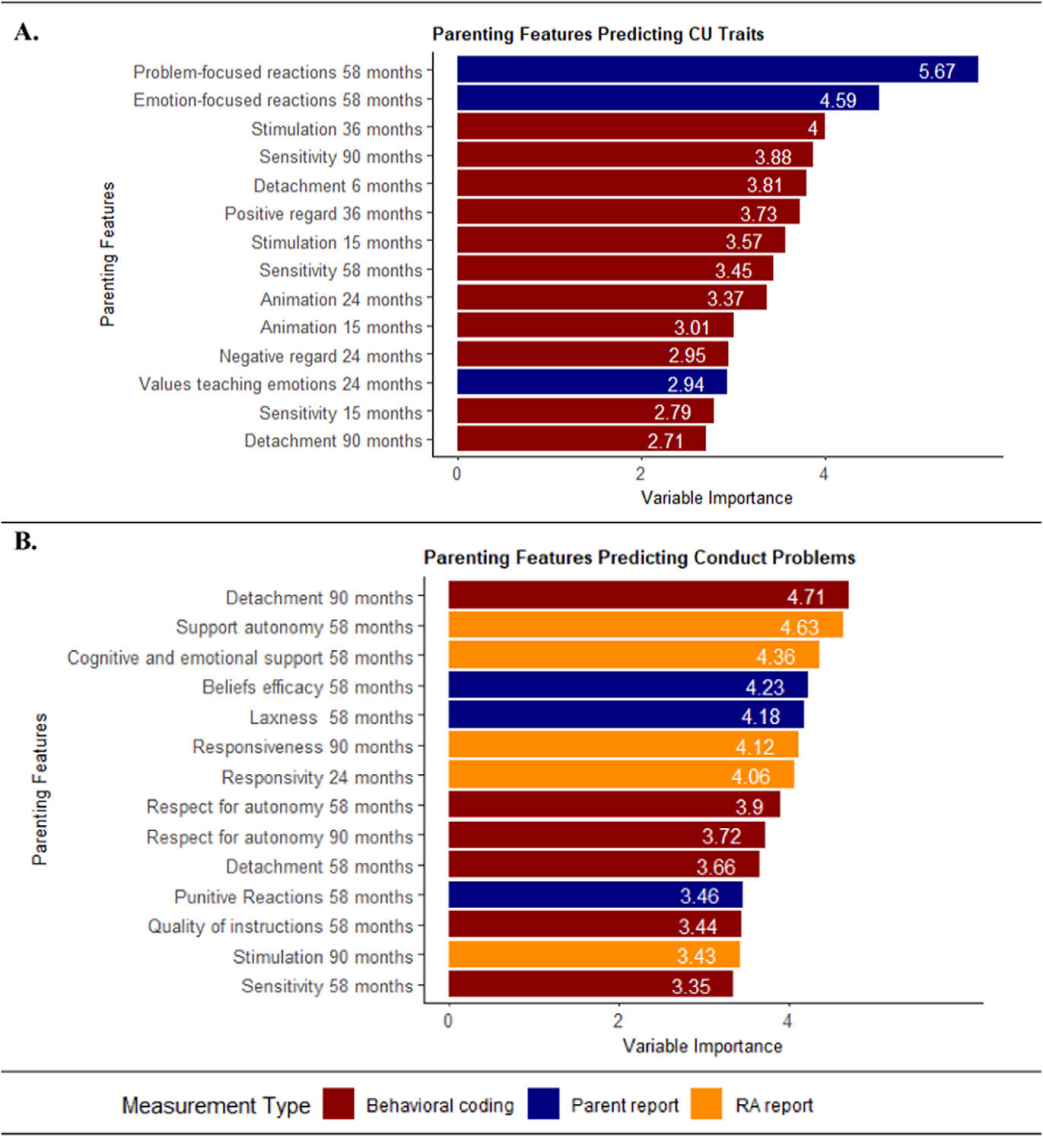
Parenting predictors categorized by measurement type. *Note*: (a) For CU traits, the full list of predictors is detailed in Supplementary Table S5. (b) For CPs, the full list of predictors is detailed in Supplementary Table S6.

**Figure 3.**
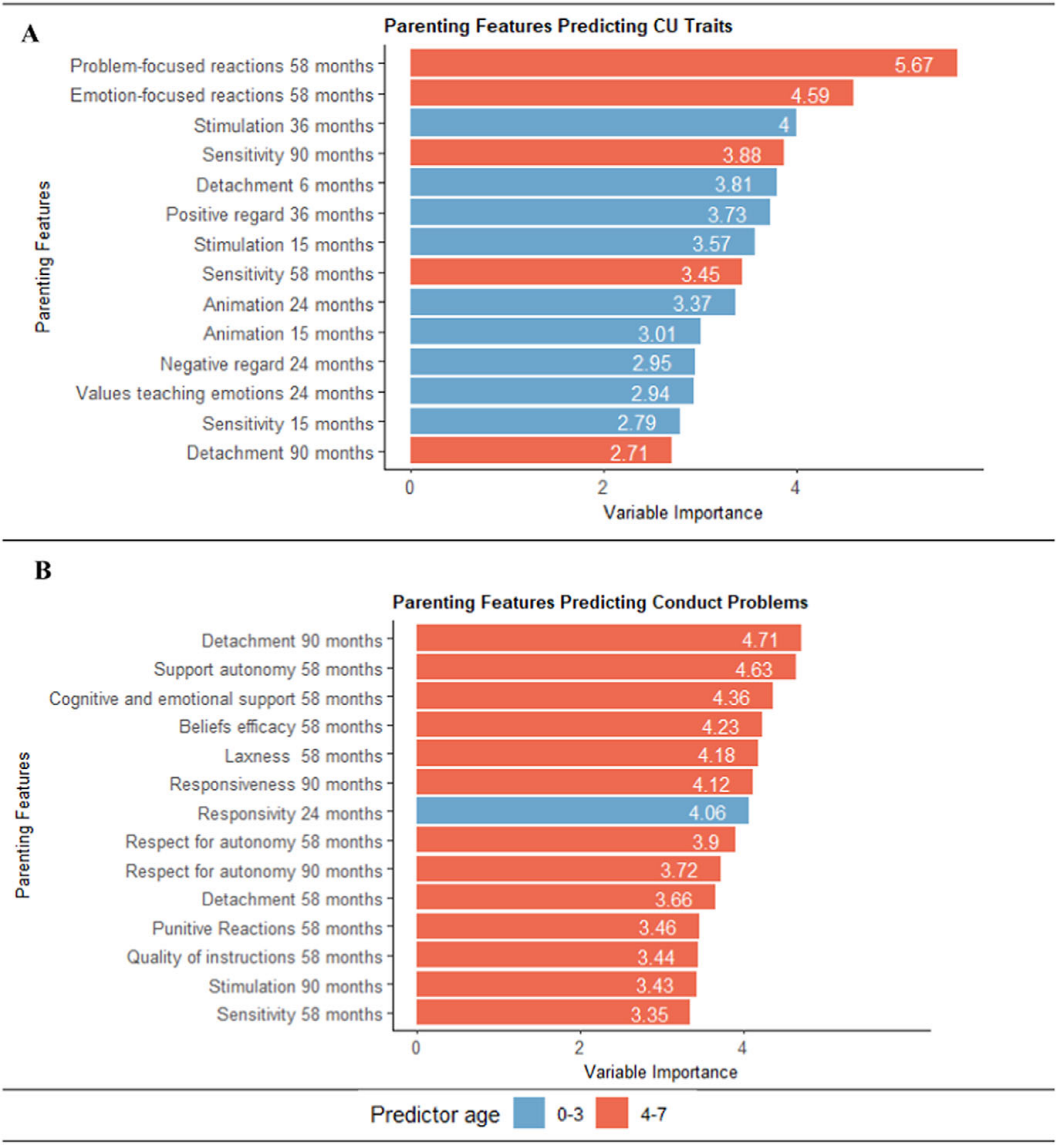
Predictors by age at assessment. *Note*: (a) For CU traits, the full list of predictors is detailed in Supplementary Table S5. (b) For CPs, the full list of predictors is detailed in Supplementary Table S6.

**Figure 4.**
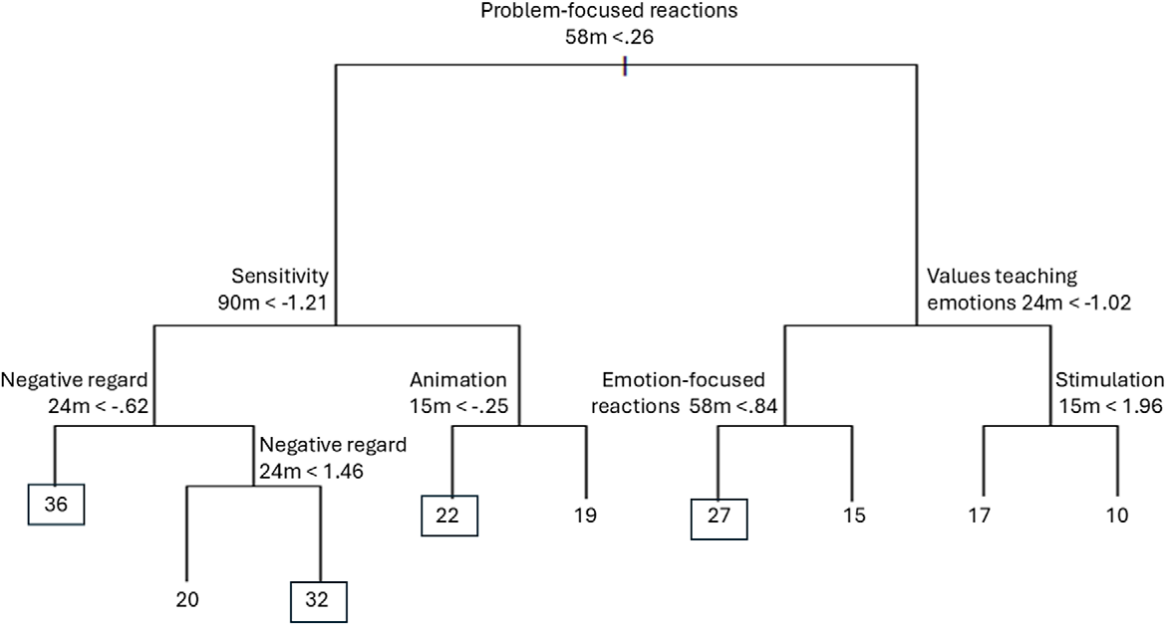
Example tree for predicting dimensional CU traits. *Note:* The figure displays only one 500 trees created for the analyses and, therefore, does not represent all trees in the forest and cannot be used in isolation for any substantive interpretation. Decision points are given as SD. The values for CU traits highlighted with a square around them are those values that fall above a recently established clinical cutoff for the measure of CU traits (Kemp et al., 2023).

Across the top 14 predictors of CU traits (Supplementary Figure S3a), most (*n =* 10, 71%) were parental emotional sensitivity (e.g. detachment and positive regard), with other predictors classified as behavior management or scaffolding The majority were behaviorally coded (*n =* 11, 79%; Figure 2a) and assessed in the first 3 years of life (*n =* 9, 64%; Figure 3a). Finally, most predictors were classified as positive parenting (*n =* 11, 79%) (Supplementary Figure S3a). Results were similar, though attenuated, using residualized CU trait scores at age 12 years (Supplementary Figure S5). For illustrative purposes, we present an example regression tree (Figure 4). Bivariate correlations showed that most top predictors (*n =* 13, 93%) were correlated with CU traits, though the magnitude of associations was modest (|*r|*’s range = .09–.22) (Supplementary Table S5). The directionality of associations was consistent with theory, indicating that less positive parenting (e.g. sensitivity) and greater negative parenting (e.g. detachment) correlate with higher CU traits.

### Predicting CPs from parenting features

We repeated the same steps for CPs. In the training sample, the best model had 72 predictors (mtry = 10), explaining 2.5% of the variability in CPs at age 12 years (SD = 2.3%). The RFE model did not differ significantly from the full model (*R* ^2^, *t*(24) = −.17, *p* = .87; RMSE: *t*(24) = .72, *p* = .48) (Supplementary Table S4). The CPs model explained significantly less variability than the CU traits model (*Z* = -4.69, *p* < .001). In the testing sample, the model explained 4.5% of the variance in CPs. As before, we focus on the top 20% of predictors for interpretability (*n =* 14) (Supplementary Table S6 presents the full list).

Across the top predictors of CPs, a third of parenting features (*n =* 5, 36%) were classified as emotional sensitivity (e.g. responsiveness), a third (*n =* 5, 36%) were behavior management (e.g. punitive reactions), and the rest were scaffolding (e.g. stimulation; *n =* 3, 21%) or lack of parental mastery (e.g. beliefs efficacy; *n =* 1, 7%) (Figure 3b). All assessment methods were represented, including research assistant report (*n =* 5, 36%), behavioral coding *(n* = 6, 43%), and parent report (*n =* 3, 21%) (Figure 2b). Most predictors were assessed during preschool (4–7 years; Figure 3b). Two-thirds were positive parenting (*n =* 9, 64%) (Supplementary Figure S3b). Only two predictors (sensitivity at age 58 months and detachment at age 90 months) were shared with the CU traits model. Bivariate correlations showed that only 21% of the top predictors and 11% of all predictors had significant linear associations, with modest effect sizes (|*r|*’s range = .10–.13; Supplementary Table S6). For one association, the direction was unexpected, with greater parental support of child autonomy at 58 months (i.e. positive parenting) associated with higher CPs (*r* = .13, *p* = .002).

## Discussion

We used ML to identify parenting features from across early childhood that predicted later CU traits and CPs. Our model explained 8.2% of the variance in CU traits. Given the temporal separation between measures (i.e. parenting at ages 6–90 months and CU traits during preadolescence), our results emphasize the importance of early parenting in shaping risk for CU traits across childhood. However, the amount of variance explained was modest, though consistent with the length of follow-up. For context, longitudinal studies with shorter follow-up periods (i.e. 2 years) explain around 20–30% of variance (e.g. Muratori et al., 2016; Waller et al., 2012), while a meta-analysis examining parenting interventions and CU traits found an effect of 1% variance explained (Perlstein et al., 2023). Thus, while the effect sizes are modest by traditional standards, they are consistent with the literature and remain meaningful, especially since even modest effects accumulate across individuals and over time to shape developmental outcomes (Funder & Ozer, 2019). Nevertheless, with the large amount of unexplained variance, our findings align with work emphasizing the importance of other factors that interact with parenting to increase risk for CU traits, including genetic risk (Takahashi, Pease, Pingault, & Viding, 2020; Viding et al., 2005), neurophysiology (Wagner et al., 2019; Wagner & Waller, 2020), temperament (Dargis & Li, 2020; Waller & Wagner, 2019), and socioeconomic factors (Carroll, Klump, & Burt, 2023; Piotrowska, Stride, Croft, & Rowe, 2015; Tomlinson et al., 2022).

Despite the exploratory nature of the analyses, we provide a data-driven approach that complements prior theory-driven efforts (Yarkoni & Westfall, 2017). In line with hypotheses, our top predictors captured emotionally sensitive parenting and were assessed in the first 3 years of life, as early as 6 months old. At age 2 years, parents’ valuing teaching about emotions was an influential predictor. In addition, behavior management difficulties emerged as an influential predictor of CU traits from 4 to 5 years old, perhaps indirect evidence of the fact that children high on CU traits would already be showing more CPs by this age. Overall, our findings align with meta-analytic work emphasizing the importance of teaching parents behavior management strategies within interventions to reduce child CPs (Gardner, Gardner, Burton, & Klimes, 2006; Leijten et al., 2022), which includes children with CU traits (Perlstein et al., 2023). Our findings on parent emotional sensitivity also support recently adapted treatments for CU traits, which emphasize positive relational and interpersonal interactions (Fleming et al., 2022). A key takeaway remains the importance of early parental emotional sensitivity in relation to CU traits. Indeed, warm, responsive, and nurturing parenting can increase children’s empathic abilities (Davidov & Grusec, 2006; Orlitsky et al., 2025) and motivation for social relationships (Feldman, 2012), which are processes impaired among children with CU traits (Viding & McCrory, 2019; Waller & Wagner, 2019). At the same time, engaging parents of children with CU traits in treatment and targeting parental warmth is challenging, especially when access to high-quality parenting interventions or therapists remains extremely limited for most families (Hyde, Tillem, Westerman, & Guzman, 2024; Perlstein et al., 2023) or if parents themselves share inherited risk for CU traits (Dadds et al., 2019; Waller et al., 2012). Tailoring parenting interventions to address these factors could enhance intervention responsiveness and ultimately improve outcomes for children with CU traits.

For CPs, although two of the top predictors were the same as for CU traits, the pattern of results largely differed. First, the overall prediction of CPs was poorer than for CU traits. Second, there was greater heterogeneity among the predictors, with greater representation of behavioral management and, surprisingly, scaffolding, alongside fewer emotional sensitivity-related predictors than the CU traits model. For assessment, ratings provided by research assistants who had conducted home visits were the predominant predictors of CPs but did not feature for CU traits. Third, parenting predictors of CPs were measured closer in time to preadolescence, whereas early parenting features (i.e. first 3 years of life) were more predictive of CU traits. Finally, there were significant linear associations between parenting features and CU traits, suggesting that the models captured direct effects. In contrast, most top-ranked predictors did not show linear associations with CPs, suggesting that interactions among predictors may have driven the variance explained or nonlinear patterns of association.

The range of parenting predictors identified in the model could also reflect the more heterogeneous phenotype of CPs (Fanti, 2018; Viding & McCrory, 2020), when compared with a more narrowly defined CU traits phenotype. Indeed, our CPs score combined symptoms of oppositional defiant disorder (ODD) and conduct disorder (CD), with the aim of capturing variability in disruptive behavior during this developmental period. However, because ODD and CD may follow partly distinct developmental trajectories (Kimonis et al., 2010), future research from later in adolescence is needed that distinguishes between these dimensions. Moreover, there are multiple theorized pathways to CPs, including those involving difficulties in emotion regulation (Marsee & Frick, 2007; Raine et al., 2006) and environmental adversity (Shaw, Shelleby, & Shaw, 2014), as well as CU traits (Frick et al., 2014; Waller et al., 2020). That is, there are likely distinct sets of parenting features that differentially increase risk for different profiles of CPs, with low parental sensitivity potentially being more strongly related to CPs characterized by uncaring or callousness (Goulter et al., 2020; Waller et al., 2014), but a lack of scaffolding being related to CPs characterized by regulation difficulties (Foley, 2017).

Our CPs model also evidenced more parenting predictors derived from research assistant ratings. This finding could reflect the centrality of broader environmental risk factors (e.g. poverty and chaos) previously linked to the development of CPs (Mills-Koonce et al., 2016; Shaw et al., 2014), which may have inadvertently shaped home visitors’ ratings. Notably, we found unexpected nonlinear and positive associations between CPs and parenting features focused on supporting child autonomy, which we had classified as “positive parenting.” This finding may reflect the balance that parents face between encouraging children’s self-expression and maintaining boundaries (i.e. in this case, closer to “permissiveness”) (Grusec & Davidov, 2010). Finally, testing the more traditional valence-based classification of parenting (“positive” vs. “negative”) was less informative than a more theoretically granular approach to characterize parenting features that could be targeted in interventions.

Our findings should be considered alongside several limitations. First, our sample size was large for a longitudinal sample, yet relatively small for ML analyses (van der Ploeg et al., 2014). Second, we had initially planned to test whether there were age-specific effects of different parenting. However, while our longitudinal dataset offered rich parenting assessments across early childhood, our design was not fully balanced, with some constructs (e.g. behavior management) measured only at particular ages. Thus, we could not rigorously evaluate when different parenting dimensions were most influential in shaping CU traits and CPs. Future studies need harmonized and consistent longitudinal designs with repeated assessments of the same constructs at multiple time points to answer both “what” and “when,” generating insights for improving the timing and targets of parenting interventions. Third, our approach precluded an examination of child-directed effects or the role of shared genetic risk factors (Pezzoli et al., 2024). Fourth, although our interpretation of findings was guided by established parenting constructs, not all the measures aligned with our theoretical framework (see Menashe-Grinberg & Atzaba-Poria, 2017; Rodrigues et al., 2024). Finally, ML methods are effective for prediction (Jiang, Gradus, & Rosellini, 2020), but are inherently “black box” approaches in the averaging of regression trees (Goh et al., 2024). Thus, future research needs to build on these findings to uncover specific interactions or mechanisms linking parenting to CU traits and CPs.

In sum, our results offer some innovative insights and clinical implications. Through the application of advanced methodological approaches, we addressed statistical limitations that have hindered a comprehensive examination of the associations between different parenting features and the emergence of CU traits and CPs, including evaluating distinct domains of parenting across distinct time windows. Findings underscore the importance of parenting practices in relation to the development of CU traits, emphasizing the need for universal screening methods to identify and support parents experiencing specific difficulties with early parental emotional sensitivity and behavior management.

## Supporting information

10.1017/S0033291726103213.sm001 Paz et al. supplementary material Paz et al. supplementary material

## Supplementary material

The supplementary material for this article can be found at http://doi.org/10.1017/S0033291726103213.
