# Supplementary material for: Using machine learning to identify parenting features prospectively related to callous-unemotional traits from infancy to early adolescence
Source: Psychol Med. 2026 Feb 18;56:e47. doi: 10.1017/S0033291726103213 (PMC12916225; doi:10.1017/S0033291726103213)
Supplement: Paz et al. supplementary material [file S0033291726103213sup001.docx]

**Supplementary Material**

**Using Machine Learning to Identify Parenting Features Prospectively Related to Callous-Unemotional Traits From Infancy to Early Adolescence**

**Supplemental Methods**

**Parenting predictors**

We screened all parenting assessments collected in the Family Life Project between ages 6 to 90 months. Every measure assessing parents' behaviors, beliefs, or opinions, using both observations and reports, was included in the current report. When a coding scheme or questionnaire included both subscales and composites, only the subscales were included. For example, the behavioral coding scheme used in the Family Life Project included a positive parenting composite consisting of the subscales of sensitivity, detachment (reversed), animation, and stimulation; the current analyses include the subscales but not the positive parenting composite. Our screening resulted in 76 different parenting features, detailed in **Table S2**. Out of the 76 features, 46 were coded from parent-child interactions, 13 were reported by the research assistant conducting the visit using the HOME observation measure, and 17 predictors were self-reported by the parent across 6 different questionnaires. For each of the 17 reported subscales, the number of items and Cronbach’s alpha are reported in **Table S1**. Most subscales showed good reliability (α > .70) and three subscales had an acceptable reliability (Over reactivity, α=.69; Value teaching emotions, α=.65; Developmental beliefs, α=.59, scale contains 3 items). Two subscales had inadequate reliability (Emotional language, α=.44 and Verbosity, α=.33) and were excluded from analyses.

| **Table S1: Sample Descriptives** | | | | | | | | | | | |
| --- | --- | --- | --- | --- | --- | --- | --- | --- | --- | --- | --- |
|  | Full FLP (*N*=1292) | Have T8 (*N*=861) | Final sample (*N*=792) | Training sample (*N*=596) | Testing sample (*N*=196) | Have T8 vs. Don’t have (861 vs. 431) | | Low vs. High missingness (792 vs. 69) | | Training vs. testing  (596 vs. 196) | |
|  | M (*SD*)/% | M (*SD*)/% | M (*SD*)/% | M (*SD*)/% | M (*SD*)/% | *t*/*χ*^2^ | *p* | *t*/*χ*^2^ | *p* | *t*/*χ*^2^ | *p* |
| Age T1 (months) | 7.74 (1.48) | 7.67 (1.47) | 7.64 (1.44) | 7.67 (1.53) | 7.57 (1.34) | 2.22 | .03 | -2.4 | .02 | -.82 | .42 |
| Age T8 (months) | 158.4 (7.53) | | 158.0 (7.28) | 158.0 (7.07) | 158.0 (7.9) | - | - | -5.9 | <.001 | .71 | .48 |
| Sex (% female) | 49% | 49% | 49% | 49% | 49% | 0 | 1.0 | .16 | .69 | 0 | 1.0 |
| Race (% Black) | 43% | 44% | 43% | 44% | 42% | 1.63 | .20 | 1.73 | .19 | .47 | .79 |
| Average InR | 1.84 (1.34) | 1.86 (1.36) | 1.60 (1.34) | 1.90 (1.35) | 1.84 (1.42) | -.90 | .37 | 1.63 | .10 | -.45 | .65 |
| Low income-to-needs ratio (% poverty) | 28% | 26% | 25% | 25% | 27% | 7.30 | .01 | 2.44 | .12 | .11 | .74 |
| State (% NC) | 60% | 57% | 55% | 57% | 54% | 7.42 | .01 | 5.29 | .02 | .47 | .49 |
| CU traits | 18.82 (9.76) | | 18.66 (9.72) | 18.7 (9.81) | 18.4 (9.48) | - | - | 1.53 | .12 | -.36 | .72 |
| CP | 5.67 (4.83) | | 5.65 (4.74) | 5.55 (4.71) | 5.96 (4.79) | - | - | .53 | .60 | 1.05 | .29 |
| CU - callous-unemotional, CP - Conduct problems. Out of the final sample of 792 children, 506 participants (64%) had complete cases with all 76 parenting predictors. Out of the remaining 272, 8 participants were missing more than one visit, 129 missed a single visit, and 135 were missing one or two observations or questionnaires despite attending all visits. | | | | | | | | | | | |

| **Table S2. Summary of parenting predictors** | | | | | | | | |
| --- | --- | --- | --- | --- | --- | --- | --- | --- |
| **#** | **Name** | **Definition** | **Type** | **Tool & coding scheme** | **Age (months)** | **Scale** | **Theoretical construct** | **Valence** |
| 1 | Sensitivity | Parent attuned, observant & responsive to child cues (ICC .75-.88) | BC | PCX (Cox, 2008) | 6,15,24,35, 58,90 | 1-5 (ages 6,15) or 1-7 (ages 24+) | Emotional sensitivity | Pos |
| 2 | Intrusiveness | Parent imposes agenda on child despite signals they need different interaction (ICC .71-.80) | BC |  | 6,15,24,35 | 1-5 (ages 6,15) or 1-7 (ages 24+) | Emotional sensitivity | Neg |
| 3 | Detachment | Parent emotionally uninvolved or disengaged & unaware of child's needs (ICC .74-.85) | BC |  | 6,15,24,35,58,90 | 1-5 (ages 6,15) or 1-7 (ages 24+) | Emotional sensitivity | Neg |
| 4 | Positive regard | Parent’s positive feelings toward the child, expressed during interaction (ICC .77-.89) | BC |  | 6,15,24,35,58,90 | 1-5 (ages 6,15) or 1-7 (ages 24+) | Emotional sensitivity | Pos |
| 5 | Negative regard | Parent’s negative feelings toward the child, expressed during interaction (ICC .75-.85) | BC |  | 6,15,24,35,58,90 | 1-5 (ages 6,15) or 1-7 (ages 24+) | Emotional sensitivity | Neg |
| 6 | Stimulation | Parent tries to foster child development & engage child in learning activities (ICC .75-.87). | BC |  | 6,15,24,3558 | 1-5 (ages 6,15) or 1-7 (ages 24+) | Scaffolding | Pos |
| 7 | Animation | Parent animated, energetic & interested in the child (ICC .76-.78). | BC |  | 6,15,24,35 | 1-5 (ages 6,15) or 1-7 (ages 24+) | Emotional sensitivity | Pos |
| 8 | Quality of instructions | Parent structures the situation so that the child knows what the task objectives & receives appropriate feedback (ICC = 80) | BC |  | 58 | 1-7 | Scaffolding | Pos |
| 9 | Respect for autonomy | Parent acts in a way that recognizes and respects the validity of the child's individuality, motives, & perspectives in the interaction (ICC =.84) | BC |  | 58,90 | 1-7 | Behavior management | Pos |
| 10 | Boundary dissolution | Parent solicits inappropriate attention, affection, intimacy, or support from child (ICC =.79). | BC |  | 58 | 1-7 | Behavior management | Neg |
| 11 | Quality of relationship | The interaction has mutual engagement between parent & child (ICC =.89). | BC |  | 58 | 1-7 | Emotional sensitivity | Pos |
| 12 | MM comments | Number of comments made by the parent relating to children’s mind during the interaction | BC | PCX | 6 | 0-22 | Emotional sensitivity | Pos |
| 13 | MM positive | Number of positive valence comments | BC |  | 6 | 0-5 | Emotional sensitivity | Pos |
| 14 | MM negative | Number of negative valence comments | BC |  | 6 | 0-7 | Emotional sensitivity | Neg |
| 15 | Responsivity | Parents being attentive, communicative & affective to the child (11 items) | RAR | HOME infancy, Caldwell & Bradley, 1984 | 6,15,24,35 (preschool version) | Sum of 0-1 questions | Emotional sensitivity | Pos |
| 16 | Acceptance | Lack of hostile or negative behaviors of the parents toward the child (8 items) | RAR |  | 6,15,24,35 (preschool version) | Sum of 0-1 questions | Emotional sensitivity | Pos |
| 17 | Support cognitive & emotional development | Positive behaviors & offering of cognitive stimulation (7 items) | RAR | HOME, element, Caldwell & Bradley, 1984 | 58 | Sum of 0-1 questions | Scaffolding | Pos |
| 18 | Support autonomy | Child can show negative affect & encouraged to make choices (4 items) | RAR |  | 58 | Sum of 0-1 questions | Behavior management | Pos |
| 19 | Responsiveness | Parent praise & encouragement of child expression (11 items) | RAR | HOME, 1^st^ grade, Caldwell & Bradley, 1984 | 90 | Sum of 0-1 questions | Emotional sensitivity | Pos |
| 20 | Stimulation | Nurturing & encouraging child learning & curiosity (14 items) | RAR |  | 90 | Sum of 0-1 questions | Scaffolding | Pos |
| 21 | Harshness | Parent expression of negativity or hostility to child (4 items) | RAR |  | 90 | Sum of 0-1 questions | Emotional sensitivity | Neg |
| 22 | Teaching emotions | Parent values teaching about emotions (22 items, α = .65) | PR | PBAF  (Dunsmore & Karn, 2001) | 24 | 1-6 | Emotional sensitivity | Pos |
| 23 | Emotional language | Parent emphasizes teaching the use of emotion language (6 items, α =.44) | PR |  | 24 | 1-6 | Emotional sensitivity | Pos |
| 24 | Developmental Beliefs | Parent belief that their child is too young to control or discuss their feelings (3 items, α =.59) | PR |  | 24 | 1-6 | Emotional sensitivity | Pos |
| 25 | Laxness | Parent’s permissive discipline (11 items, α =.73, .74) | PR | Parenting scale  (Arnold et al., 1993) | 48,58 | 1-7 | Behavior management | Neg |
| 26 | Over reactivity | Parent’s anger, meanness & irritability toward the child (10 items, α =.69) | PR |  | 48 | 1-7 | Behavior management | Neg |
| 27 | Verbosity | Reliance on talking even when talking is ineffective (7 items, α =.33) | PR |  | 48 | 1-7 | Behavior management | Neg |
| 28 | Beliefs spoiling | Parent’s belief children can be spoiled by responsiveness and affection (7 items, α =.88) | PR | PBS  (Luster, 1985) | 58 | 1-6 | Emotional sensitivity | Neg |
| 29 | Beliefs efficacy | Parent’s disbelief regarding environmental influence of child development (7 items, α =.79). | PR |  | 58 | 1-6 | Parental mastery | Neg |
| 30 | Punitive Reactions | Parents respond with punitive reactions that decrease their need to deal with the negative emotions of their children (12 scenarios, α =.82) | PR | CCNES (Fabes et al., 1990) | 58 | 1-5 | Behavior management | Neg |
| 31 | Minimization Reactions | Parent minimizes seriousness  of the situation or devalues child's distress (12 scenarios, α =.81) | PR |  | 58 | 1-5 | Behavior management | Neg |
| 32 | Emotion-Focused Reactions | Parent responds with  strategies that are designed to help the child feel better (12 scenarios, α =.86) | PR |  | 58 | 1-5 | Behavior management | Pos |
| 33 | Problem-Focused Reactions | Parent helps child  solve the problem that caused the child's distress (12 scenarios, α =.81) | PR |  | 58 | 1-5 | Behavior management | Pos |
| 34 | Adult control | Perceived controllability of the adult's contribution to caregiving failure (6 items, α =.86) | PR | PAT (Bugental, 1987) | 58 | 0-6 | Parental mastery | Pos |
| 35 | Child control | Perceived controllability of the child's contribution to caregiving failure (6 items, α =.83) | PR |  | 58 | 0-6 | Parental mastery | Neg |
| 36 | Uncontrollable success | Perceived uncontrollability of the adult's contribution to success (6 items, α =.80) | PR |  | 58 | 0-6 | Parental mastery | Neg |
| 37 | Perceived control | Ratio between perceived adult & child control (variables 34 and 35) | PR |  | 58 | 0-6 | Parental mastery | Pos |
| MM mind-mindedness. BC – Behavioral coding; RAR – Research assistant report; PR – Parent report. PCX – Parent child interaction; PBAF – Parent’s Beliefs About Emotions. POS – Parent’s Opinion Survey. CCNES – Coping with Children’s Negative Emotion Scale. PAT – Parent’s Attribution Test. Pos – positive parenting, Neg – negative parenting. | | | | | | | | |

| **Table S3: Within-variable correlations of repeated parenting assessments across time** | | | | | |
| --- | --- | --- | --- | --- | --- |
| **#** | **Parenting predictor** | **N of time points** | **Minimum correlation** | **Maximum correlation** | **Mean correlation** |
| 1 | Sensitivity | 6 | 0.36 | 0.65 | 0.48 |
| 2 | Intrusiveness | 4 | 0.24 | 0.51 | 0.32 |
| 3 | Detachment | 6 | 0.34 | 0.52 | 0.42 |
| 4 | Positive regard | 6 | 0.38 | 0.56 | 0.46 |
| 5 | Negative regard | 6 | 0.19 | 0.44 | 0.32 |
| 6 | Stimulation | 6 | 0.28 | 0.55 | 0.43 |
| 7 | Animation | 4 | 0.41 | 0.54 | 0.48 |
| 8 | Respect for autonomy | 2 | 0.57 | 0.57 | 0.57 |
| 9 | Responsivity | 4 | 0.18 | 0.37 | 0.24 |
| 10 | Acceptance | 4 | 0.08 | 0.42 | 0.28 |
| 11 | Laxness | 2 | 0.60 | 0.60 | 0.60 |
| Correlations were calculated for all variables that were measured repeatedly for more than one time | | | | | |

| **Table S4.** Model results across full and RFE models and t-tests reporting no significant differences in performance. | | | | | | |
| --- | --- | --- | --- | --- | --- | --- |
| **CU Traits** | | | | | | |
|  | Training Data | | Testing Data | | Model Differences (training) | |
|  | Full Model (74 features) | Post-RFE Model (71 features) | Full Model (74 features) | Post-RFE Model (71 features) | *t* | *p* |
| mtry | 5 | 5 |  |  |  |  |
| R^2^ | 7.07% | 6.65% | 7.54% | 8.17% | .041 | .684 |
| RMSE | 9.49 | 9.51 | 9.10 | 9.07 | -.12 | .904 |
| **CP** | | | | | | |
|  | Training Data | | Testing Data | | Model Differences (training) | |
|  | Full Model (74 features) | Post-RFE Model (72 features) | Full Model (74 features) | Post-RFE Model (71 features) | *t* | *p* |
| mtry | 50 | 10 |  |  |  |  |
| R^2^ | 2.37% | 2.48% | 5.28% | 4.54% | -.17 | .869 |
| RMSE | 4.71 | 4.68 | 4.66 | 4.68 | .72 | .476 |

| **Table S5** Machine Learning Model Predicting CU traits | | | | | | | | |
| --- | --- | --- | --- | --- | --- | --- | --- | --- |
| **Rank** | **Predictor** | **Age (m)** | **Type** | **Theoretical construct** | **Valence** | **Variable importance** | ***r*** | ***p*** |
| 1 | Problem-focused reactions | 58 | PR | BM | Pos | 5.67 | -.19 | <.001 |
| 2 | Emotion-focused reactions | 58 | PR | BM | Pos | 4.59 | -.16 | <.001 |
| 3 | Stimulation | 35 | BC | SC | Pos | 4.00 | -.12 | .003 |
| 4 | Sensitivity | 90 | BC | ES | Pos | 3.88 | -.20 | <.001 |
| 5 | Detachment | 6 | BC | ES | Neg | 3.81 | .09 | .022 |
| 6 | Positive regard | 35 | BC | ES | Pos | 3.73 | -.15 | <.001 |
| 7 | Stimulation | 15 | BC | SC | Pos | 3.57 | -.22 | <.001 |
| 8 | Sensitivity | 58 | BC | ES | Pos | 3.45 | -.18 | <.001 |
| 9 | Animation | 24 | BC | ES | Pos | 3.37 | -.06 | .118 |
| 10 | Animation | 15 | BC | ES | Pos | 3.01 | -.17 | <.001 |
| 11 | Negative regard | 24 | BC | ES | Neg | 2.95 | .14 | <.001 |
| 12 | Values teaching emotions | 24 | PR | ES | Pos | 2.94 | -.18 | <.001 |
| 13 | Sensitivity | 15 | BC | ES | Pos | 2.80 | -.20 | <.001 |
| 14 | Detachment | 90 | BC | ES | Neg | 2.71 | .19 | <.001 |
| 15 | Minimization Reactions | 58 | PR | BM | Neg | 2.70 | .15 | <.001 |
| 16 | Detachment | 15 | BC | ES | Neg | 2.70 | .22 | <.001 |
| 17 | Responsiveness | 90 | RAR | ES | Pos | 2.69 | -.14 | <.001 |
| 18 | Respect for autonomy | 58 | BC | BM | Pos | 2.56 | -.18 | <.001 |
| 19 | Animation | 6 | BC | ES | Pos | 2.55 | -.08 | .050 |
| 20 | Sensitivity | 6 | BC | ES | Pos | 2.53 | -.10 | .020 |
| 21 | Beliefs efficacy | 58 | PR | PM | Neg | 2.50 | .15 | <.001 |
| 22 | Animation | 35 | BC | ES | Pos | 2.37 | -.11 | .008 |
| 23 | Child control | 58 | PR | PM | NR | 2.28 | .08 | .045 |
| 24 | Beliefs spoiling | 58 | PR | ES | Neg | 2.20 | .16 | <.001 |
| 25 | Over reactivity | 48 | PR | BM | Neg | 2.17 | .17 | <.001 |
| 26 | Positive regard | 6 | BC | ES | Pos | 2.11 | -.09 | .032 |
| 27 | Stimulation | 90 | RAR | SC | Pos | 2.09 | -.16 | <.001 |
| 28 | Responsivity | 15 | RAR | ES | Pos | 1.81 | -.15 | <.001 |
| 29 | Boundary dissolution | 90 | BC | BM | Neg | 1.71 | -.03 | .455 |
| 30 | Positive regard | 15 | BC | ES | Pos | 1.65 | -.17 | <.001 |
| 31 | Positive regard | 24 | BC | ES | Pos | 1.62 | -.15 | <.001 |
| 32 | Support cognitive and emotional development | 58 | RAR | SC | Pos | 1.62 | -.11 | .005 |
| 33 | Stimulation | 24 | BC | SC | Pos | 1.50 | -.13 | <.001 |
| 34 | Negative regard | 90 | BC | ES | Neg | 1.45 | .03 | .468 |
| 35 | Intrusiveness | 35 | BC | ES | Neg | 1.41 | .10 | .014 |
| 36 | Intrusiveness | 6 | BC | ES | Neg | 1.40 | .03 | .486 |
| 37 | Quality of instructions | 58 | BC | SC | Pos | 1.28 | -.10 | .015 |
| 38 | Sensitivity | 24 | BC | ES | Pos | 1.25 | -.12 | .005 |
| 39 | Respect for autonomy | 90 | BC | BM | Pos | 1.09 | -16 | <.001 |
| 40 | Responsivity | 6 | RAR | ES | Pos | 1.04 | -.11 | .005 |
| 41 | Detachment | 58 | BC | ES | Neg | 1.02 | .12 | .003 |
| 42 | MM positive | 6 | BC | ES | Pos | 0.98 | .05 | .261 |
| 43 | Negative regard | 90 | BC | ES | Neg | 0.96 | .03 | .468 |
| 44 | MM comments | 6 | BC | ES | Pos | 0.84 | -.06 | .120 |
| 45 | Boundary dissolution | 58 | BC | BM | Neg | 0.82 | .01 | .818 |
| 46 | Punitive Reactions | 58 | PR | BM | Neg | 0.60 | .15 | <.001 |
| 47 | Positive regard | 58 | BC | ES | Pos | 0.41 | -.16 | <.001 |
| 48 | Perceived control | 58 | PR | PM | NR | 0.40 | -.09 | .023 |
| 49 | Detachment | 24 | BC | ES | Neg | 0.19 | .11 | .005 |
| 50 | Intrusiveness | 24 | BC | ES | Neg | 0.15 | .11 | .005 |
| 51 | Acceptance | 24 | RAR | ES | Pos | 0.14 | -.11 | .009 |
| 52 | Developmental beliefs | 24 | PR | ES | Pos | 0.06 | -.08 | .054 |
| 53 | MM negative | 6 | BC | ES | Neg | 0.00 | .03 | .426 |
| 54 | Sensitivity | 35 | BC | ES | Pos | -0.07 | -.13 | .001 |
| 55 | Stimulation | 6 | BC | SC | Pos | -0.11 | -.12 | .003 |
| 56 | Negative regard | 15 | BC | ES | Neg | -0.21 | .09 | .037 |
| 57 | Negative regard | 35 | BC | ES | Neg | -0.27 | .12 | .002 |
| 58 | Negative regard | 6 | BC | ES | Neg | -0.39 | .05 | .188 |
| 59 | Uncontrollable success | 58 | PR | PM | NR | -0.46 | -.03 | .504 |
| 60 | Acceptance | 15 | RAR | ES | Pos | -0.64 | -.12 | .005 |
| 61 | Support autonomy | 58 | RAR | BM | Pos | -0.71 | -.18 | <.001 |
| 62 | Detachment | 35 | BC | ES | Neg | -0.73 | .14 | <.001 |
| 63 | Responsivity | 24 | RAR | ES | Pos | -0.83 | -.07 | .068 |
| 64 | Acceptance | 6 | RAR | ES | Pos | -0.84 | -.09 | .023 |
| 65 | Adult control | 58 | PR | PM | NR | -0.93 | -.05 | .204 |
| 66 | Acceptance | 35 | RAR | ES | Pos | -1.22 | -.09 | .026 |
| 67 | Harshness | 90 | RAR | ES | Neg | -1.32 | .04 | .336 |
| 68 | Negative regard | 58 | BC | ES | Neg | -1.51 | .08 | .059 |
| 69 | Stimulation | 58 | BC | SC | Pos | -1.77 | -.15 | <.001 |
| 70 | Responsivity | 35 | BC | ES | Pos | -1.91 | -.09 | .029 |
| 71 | Quality of relationship | 58 | BC | ES | Pos | -2.36 | -.10 | .013 |
| Type: BC = Behavioral Coding; PR = Parental report; RAR = research Assistant Report. Theoretical constructs: BM = Behavior Management; ES = Emotional Sensitivity; PM = Parental Mastery; SC = Scaffolding. Pos = Positive; Neg = Negative. To ensure that no data leakage occurred between the training and testing sets, we performed a sensitivity analysis in which missing data were imputed after the train–test split. Results were highly similar (6.5% variance explained in the training set and 9.3% in the testing set). Results remain similar when chosen model was applied on the subset of complete cases (9.9% variance explained in the training set (*N*=380) and 8.0% in the testing set (*N*=126)), confirming that the original data processing did not introduce leakage or inflate model performance. | | | | | | | | |

| **Table S6.** Machine Learning Prediction of Conduct Problems | | | | | | | | |
| --- | --- | --- | --- | --- | --- | --- | --- | --- |
| **Rank** | **Predictor** | **Age (m)** | **Type** | **Theoretical construct** | **Valence** | **Variable importance** | ***r*** | ***p*** |
| 1 | Detachment | 90 | BC | ES | Neg | 4.71 | .02 | .589 |
| 2 | Support autonomy | 58 | RAR | BM | Pos | 4.63 | .13 | .002 |
| 3 | Support cognitive and emotional development | 58 | RAR | SC | Pos | 4.36 | -.13 | .002 |
| 4 | Beliefs efficacy | 58 | PR | PM | Neg | 4.23 | -.05 | .263 |
| 5 | Laxness | 58 | PR | BM | Neg | 4.18 | .10 | .010 |
| 6 | Responsiveness | 90 | RAR | ES | Pos | 4.12 | -.08 | .057 |
| 7 | Responsivity | 24 | RAR | ES | Pos | 4.06 | -.04 | .311 |
| 8 | Respect for autonomy | 58 | BC | BM | Pos | 3.90 | -.02 | .580 |
| 9 | Respect for autonomy | 90 | BC | BM | Pos | 3.72 | -.01 | .889 |
| 10 | Detachment | 58 | BC | ES | Neg | 3.66 | -.01 | .834 |
| 11 | Punitive Reactions | 58 | PR | BM | Neg | 3.46 | .05 | .220 |
| 12 | Quality of instructions | 58 | BC | SC | Pos | 3.44 | .02 | .654 |
| 13 | Stimulation | 90 | RAR | SC | Pos | 3.43 | -.06 | .126 |
| 14 | Sensitivity | 58 | BC | ES | Pos | 3.35 | -.01 | .740 |
| 15 | Detachment | 15 | BC | ES | Neg | 3.34 | .11 | .006 |
| 16 | Negative regard | 58 | BC | ES | Neg | 3.12 | -.01 | .792 |
| 17 | Responsivity | 6 | RAR | ES | Pos | 2.95 | -.05 | .196 |
| 18 | Sensitivity | 6 | BC | ES | Pos | 2.81 | -.02 | .595 |
| 19 | Sensitivity | 90 | BC | ES | Pos | 2.68 | -.03 | .446 |
| 20 | Responsivity | 35 | RAR | ES | Pos | 2.64 | -.07 | .081 |
| 21 | Quality of relationship | 58 | BC | ES | Pos | 2.47 | -.06 | .174 |
| 22 | Positive regard | 24 | BC | ES | Pos | 2.37 | .01 | .857 |
| 23 | Boundary dissolution | 58 | BC | BM | Neg | 2.10 | .05 | .188 |
| 24 | Animation | 24 | BC | ES | Pos | 1.97 | .00 | .939 |
| 25 | Stimulation | 24 | BC | SC | Pos | 1.96 | -.05 | .215 |
| 26 | Over reactivity | 48 | PR | BM | Neg | 1.84 | .10 | .012 |
| 27 | Perceived control | 58 | PR | PM | NR | 1.62 | -.03 | .526 |
| 28 | Positive regard | 15 | BC | ES | Pos | 1.60 | -.06 | .148 |
| 29 | Acceptance | 15 | RAR | ES | Pos | 1.59 | -.06 | .153 |
| 30 | MM comments | 6 | BC | ES | Pos | 1.41 | .01 | .887 |
| 31 | Emotion-focused reaction | 58 | PR | BM | Pos | 1.41 | -.02 | .676 |
| 32 | Detachment | 24 | BC | ES | Neg | 1.38 | .03 | .454 |
| 33 | Harshness | 90 | RAR | ES | Neg | 1.14 | .01 | .850 |
| 34 | Acceptance | 24 | RAR | ES | Pos | 1.13 | -.07 | .097 |
| 35 | Acceptance | 6 | RAR | ES | Pos | 1.13 | -.02 | .703 |
| 36 | Developmental beliefs | 24 | PR | ES | Pos | 1.06 | -.04 | .344 |
| 37 | Laxness | 48 | PR | BM | Neg | 0.98 | .04 | .373 |
| 38 | Sensitivity | 24 | BC | ES | Pos | 0.95 | -.02 | .651 |
| 39 | Negative regard | 35 | BC | ES | Neg | 0.91 | .06 | .152 |
| 40 | Minimization Reactions | 58 | PR | BM | Neg | 0.87 | .05 | .253 |
| 41 | Stimulation | 15 | BC | SC | Pos | 0.76 | -.07 | .112 |
| 42 | Stimulation | 6 | BC | SC | Pos | 0.65 | -.07 | .079 |
| 43 | Child control | 58 | PR | PM | NR | 0.62 | .02 | .609 |
| 44 | Beliefs spoiling | 58 | PR | ES | Neg | 0.61 | -.03 | .469 |
| 45 | Sensitivity | 35 | BC | ES | Pos | 0.57 | .00 | .931 |
| 46 | Detachment | 6 | BC | ES | Neg | 0.51 | .06 | .122 |
| 47 | Boundary dissolution | 90 | BC | BM | Neg | 0.45 | .01 | .793 |
| 48 | Positive regard | 58 | BC | ES | Pos | 0.38 | -.03 | .420 |
| 49 | Negative regard | 15 | BC | ES | Neg | 0.34 | .02 | .575 |
| 50 | Responsivity | 15 | RAR | ES | Pos | 0.33 | -.09 | .030 |
| 51 | Stimulation | 58 | BC | SC | Pos | 0.29 | -.01 | .818 |
| 52 | Stimulation | 35 | BC | SC | Pos | 0.25 | -.07 | .107 |
| 53 | Positive regard | 6 | BC | ES | Pos | 0.21 | -.03 | .490 |
| 54 | Sensitivity | 15 | BC | ES | Pos | 0.20 | -.11 | .009 |
| 55 | Sensitivity | 90 | BC | ES | Pos | 0.03 | -.03 | .446 |
| 56 | Problem-focused reaction | 58 | PR | BM | Pos | -0.05 | .01 | .851 |
| 57 | Animation | 15 | BC | ES | Pos | -0.05 | -.08 | .039 |
| 58 | Positive regard | 35 | BC | ES | Pos | -0.15 | -.03 | .421 |
| 59 | Intrusiveness | 6 | BC | ES | Neg | -0.19 | -.03 | .448 |
| 60 | Negative regard | 24 | BC | ES | Neg | -0.30 | .03 | .437 |
| 61 | Intrusiveness | 35 | BC | ES | Neg | -0.34 | .02 | .653 |
| 62 | Negative regard | 90 | BC | ES | Neg | -0.51 | .06 | .130 |
| 63 | Animation | 35 | BC | ES | Pos | -0.56 | -.04 | .336 |
| 64 | Animation | 6 | BC | ES | Pos | -0.61 | -.03 | .412 |
| 65 | Acceptance | 35 | RAR | ES | Pos | -0.61 | -.03 | .400 |
| 66 | Uncontrollable success | 58 | PR | PM | NR | -0.64 | -.05 | .229 |
| 67 | Intrusiveness | 24 | BC | ES | Neg | -0.78 | -.01 | .753 |
| 68 | Intrusiveness | 15 | BC | ES | Neg | -0.91 | .05 | .226 |
| 69 | Values teaching emotions | 24 | PR | ES | Pos | -1.72 | -.02 | .554 |
| 70 | Adult control | 58 | PR | PM | NR | -1.82 | -.03 | .524 |
| 71 | Detachment | 35 | BC | ES | Neg | -2.05 | .06 | .153 |
| 72 | Negative regard | 6 | BC | ES | Neg | -2.14 | .02 | .671 |
| Type: BC = Behavioral Coding; PR = Parental report; RAR = research Assistant Report. Theoretical constructs: BM = Behavior Management; ES = Emotional Sensitivity; PM = Parental Mastery; SC = Scaffolding. | | | | | | | | |

**
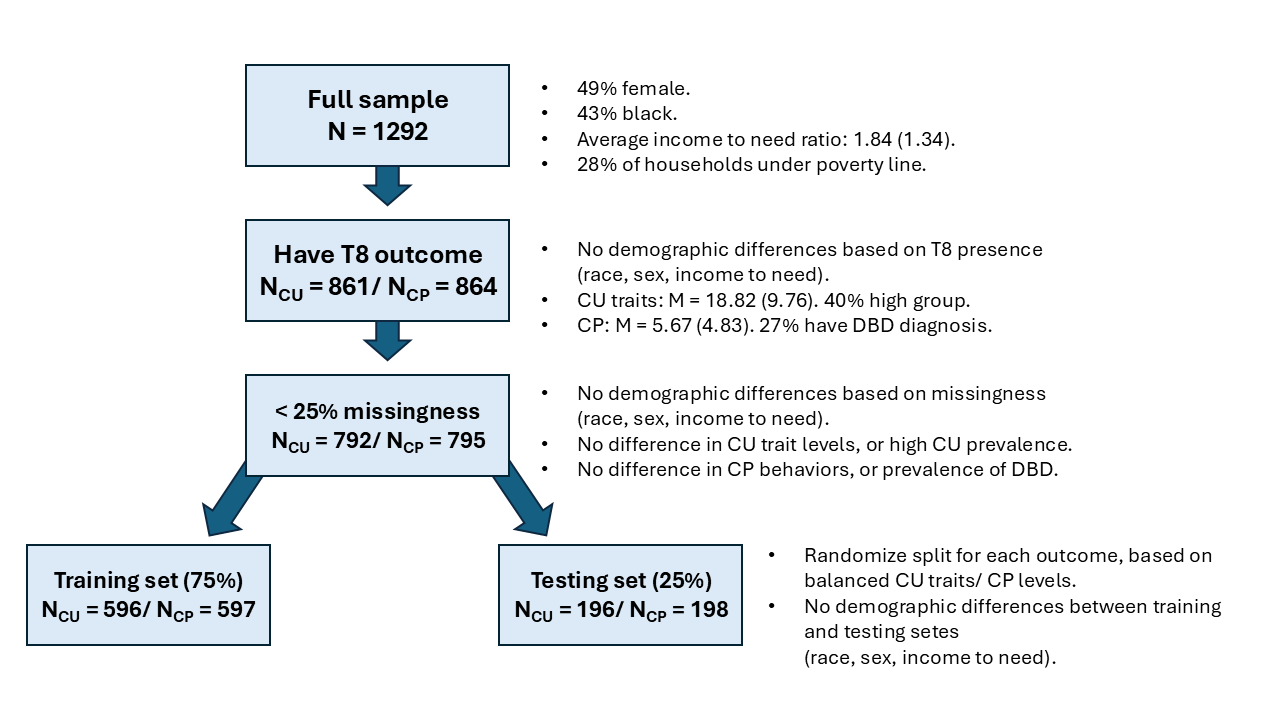
Figure S1.** Overview of Sample

**
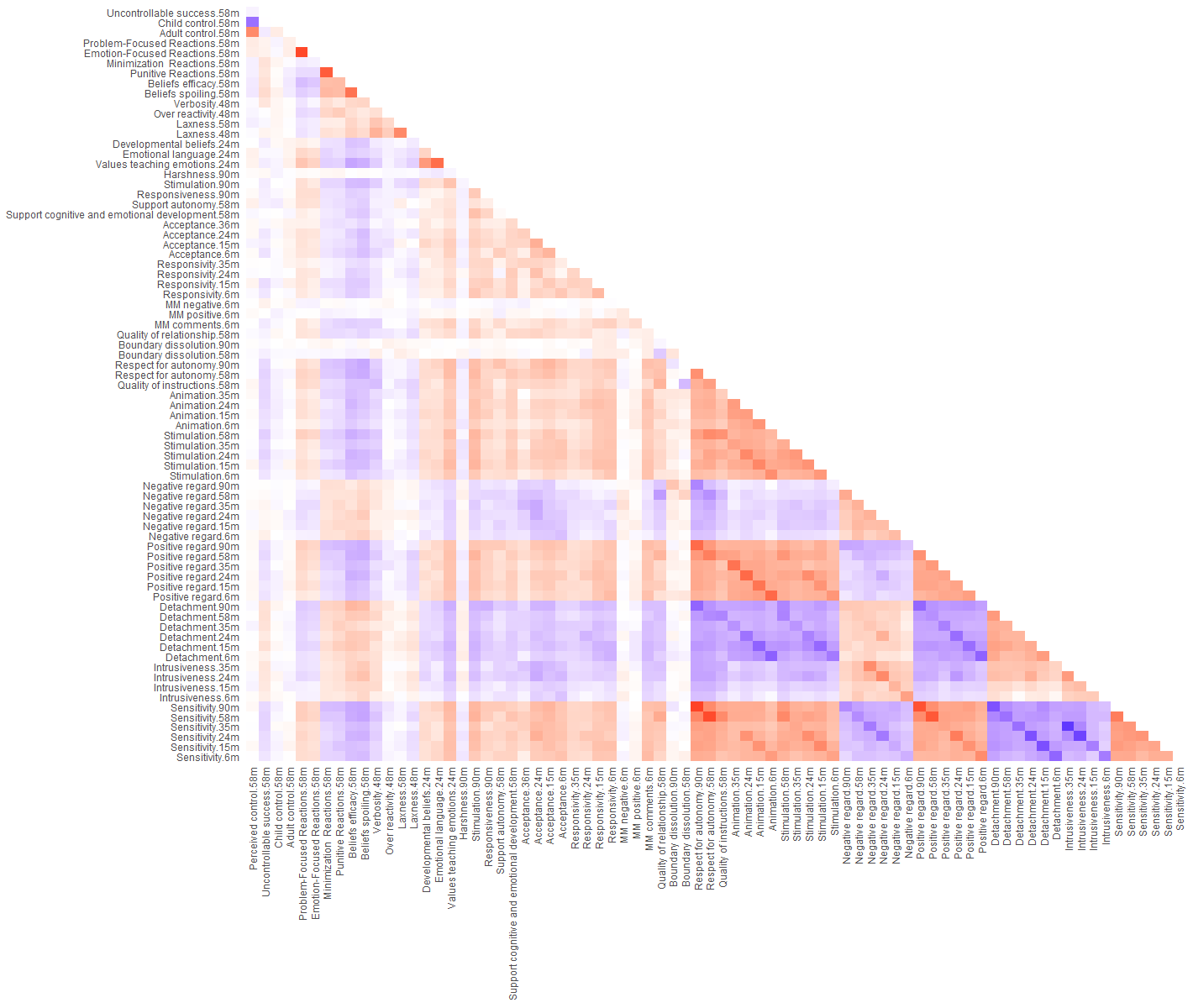
Figure S2.** Bivariate correlations between all parenting features (heat map
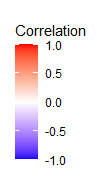
)

| **Figure S3.** Top predictors by variable importance |
| --- |
| 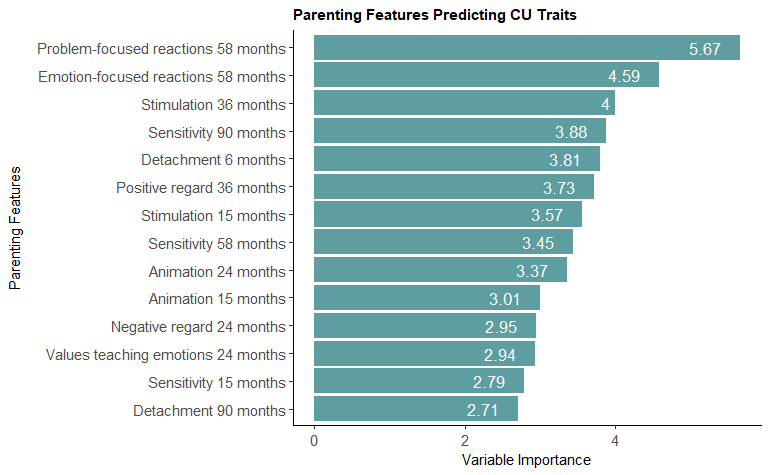  A) |
| 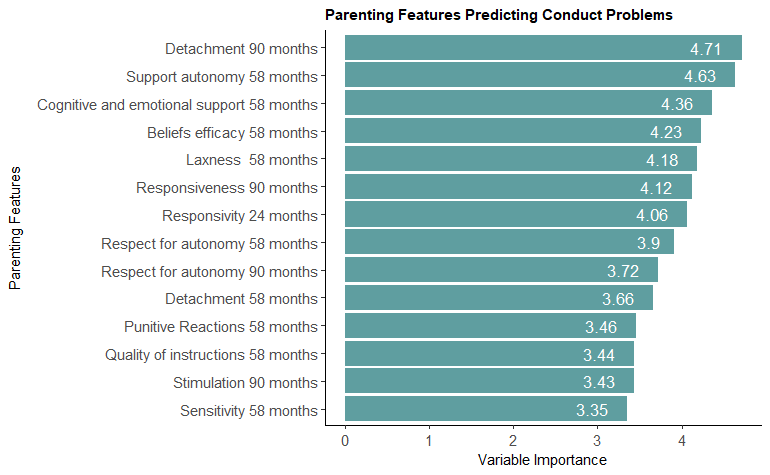  B) |
| Notes. A) For CU traits, the full list of predictors is detailed in Table S5. B) For CP, the full list of predictors is detailed in Table S6. |

| **Figure S4.** Predictors by valence of parenting behavior |
| --- |
| 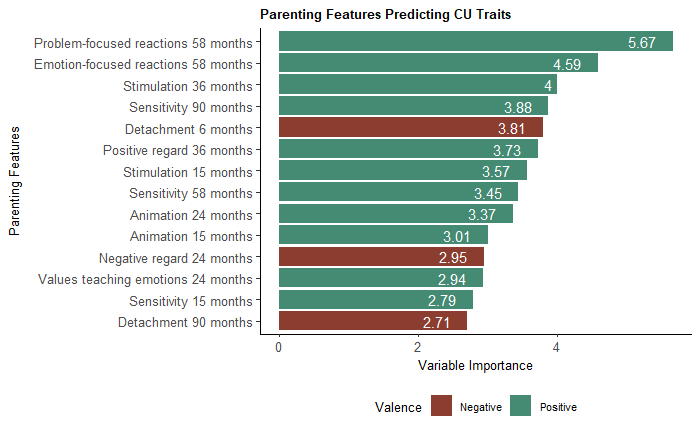  A) |
| 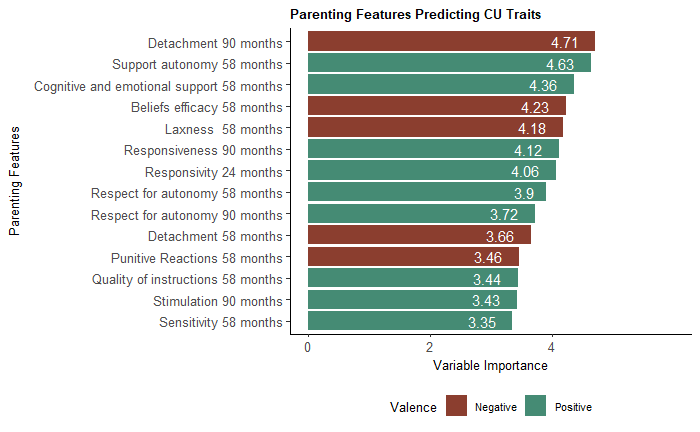  B) |
| 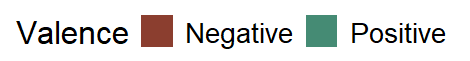 |
| Notes. A) For CU traits, the full list of predictors is detailed in Table S5. B) For CP, the full list of predictors is detailed in Table S6. |

| **Figure S5.** Top predictors by variable importance |
| --- |
| 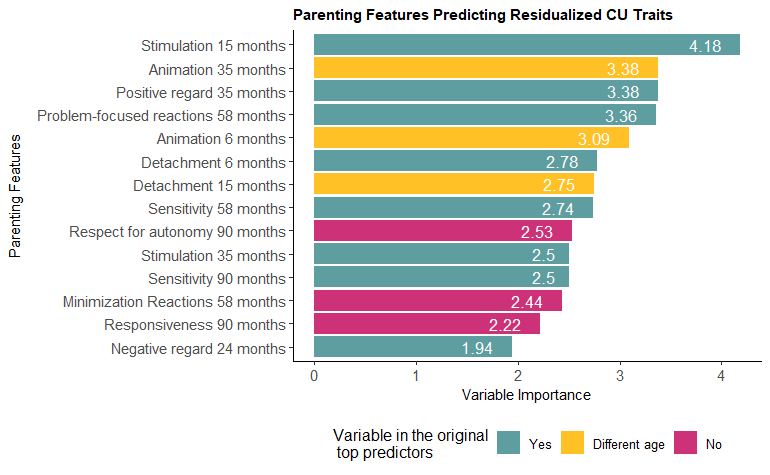  A) |
| 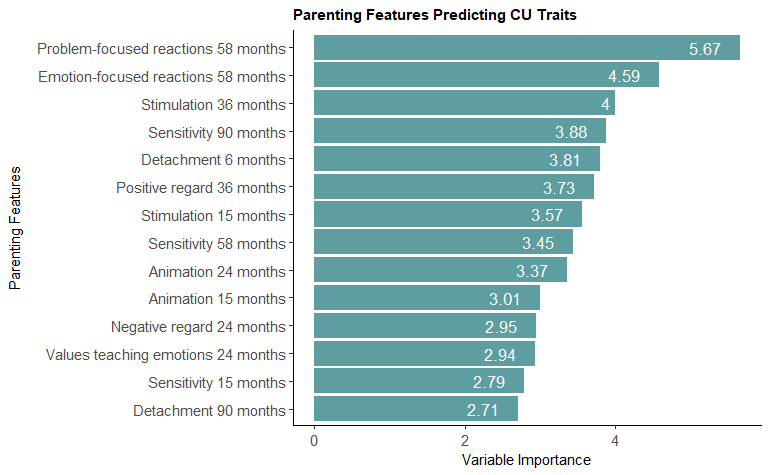  B) |
| 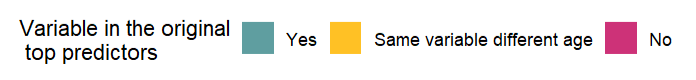 |
| Notes. Outcome was created by residualizing preadolescence CU traits on early CU traits levels from age 48 months, representing the change in construct over time. RFE found the best model included all 74 predictors and explained 3.6% of variability in the training set, and 6.4% of the variability in testing set. Figure S5a presents the top 14 predictors of the residualized outcome; 65% (*N*=8) of the predictors overlap with the original top 14 predictors of preadolescence CU traits (presented in Figure S5b), and only 21% (*N*=3) are new predictors. |
